# Ultrasonic Sensing of Plant Water Needs for Agriculture

**DOI:** 10.3390/s16071089

**Published:** 2016-07-14

**Authors:** Tomas Gómez Álvarez-Arenas, Eustaquio Gil-Pelegrin, Joao Ealo Cuello, Maria Dolores Fariñas, Domingo Sancho-Knapik, David Alejandro Collazos Burbano, Jose Javier Peguero-Pina

**Affiliations:** 1 Instituto de Tecnologías Físicas y de la Información, Spanish National Research Council (CSIC), Serrano 144, Madrid 28006, Spain; md.farinas@csic.com; 2 Unidad de Recursos Forestales, Centro de Investigación y Tecnología Agroalimentaria-Instituto Agroalimentario de Aragón (IA2), Gobierno de Aragón, Av. Montañana, 930, Zaragoza 50059, Spain; egilp@cita-aragon.com (E.G.-P.); dsancho@cita-aragon.com (D.S.-K.); jjpeguero@aragon.es (J.J.P.-P.); 3 Escuela de Ingeniería Mecánica, Ed. 351, 2011, Ciudad Universitaria- Meléndez, Universidad del Valle, Cali 760032, Colombia; joao.ealo@correounivalle.edu.co (J.E.C.); david.collazos@correounivalle.edu.co (D.A.C.B.)

**Keywords:** air-coupled ultrasound, resonant spectroscopy, non-contact sensing, non-destructive sensing, water potential, relative water content, irrigation control, 43.35.Cg, 43.35.Yb, 43.35.Zc, 43.38.Fx, 43.60.Pt, 43.60.Vx, 87.19.In, 87.19.R., 87.50.Y, 87.80.Dj, 87.80.Ek, 87.85.fk

## Abstract

Fresh water is a key natural resource for food production, sanitation and industrial uses and has a high environmental value. The largest water use worldwide (~70%) corresponds to irrigation in agriculture, where use of water is becoming essential to maintain productivity. Efficient irrigation control largely depends on having access to reliable information about the actual plant water needs. Therefore, fast, portable and non-invasive sensing techniques able to measure water requirements directly on the plant are essential to face the huge challenge posed by the extensive water use in agriculture, the increasing water shortage and the impact of climate change. Non-contact resonant ultrasonic spectroscopy (NC-RUS) in the frequency range 0.1–1.2 MHz has revealed as an efficient and powerful non-destructive, non-invasive and in vivo sensing technique for leaves of different plant species. In particular, NC-RUS allows determining surface mass, thickness and elastic modulus of the leaves. Hence, valuable information can be obtained about water content and turgor pressure. This work analyzes and reviews the main requirements for sensors, electronics, signal processing and data analysis in order to develop a fast, portable, robust and non-invasive NC-RUS system to monitor variations in leaves water content or turgor pressure. A sensing prototype is proposed, described and, as application example, used to study two different species: *Vitis vinifera* and *Coffea arabica*, whose leaves present thickness resonances in two different frequency bands (400–900 kHz and 200–400 kHz, respectively), These species are representative of two different climates and are related to two high-added value agricultural products where efficient irrigation management can be critical. Moreover, the technique can also be applied to other species and similar results can be obtained.

## 1. Introduction

World agriculture consumes approximately 70% of the fresh water withdrawn per year to irrigate only about 17% of the world’s cropland [1]. This amount of irrigated land is slowly expanding due to the increased human food requirements and the effects of global warming [1,2]. The application of right agricultural practices and supporting policy solutions is then crucial; in particular, water efficiency in crop irrigation can be largely improved by introducing more accurate systems to indicate the actual water need of the crop [3].

*Vitis vinifera* is evolutionarily well-suited to dry climates, but prolonged water scarcity or fluctuating water soil availability severely affects berry quality [4], reduces yield and compromises economic viability of the crop. Increasing water scarcity could lead to a more frequent use of irrigation for an affordable crop production [4] but, generous watering can reduce the quality of the fruit, through a decrease in colour and sugar content, and can imbalance the acidity and interfere with the flavonoid development [5]. Due to the large dependence of berry quality parameters on soil water availability, irrigation should be accurately regulated through the development of new methods of accurate irrigation scheduling based on plant “stress sensing” to achieve a more environmentally sustainable viticulture with a reasonable fruit quality [6,7,8].

Coffee is a traditional and widely consumed beverage in many countries and has a high impact in the economical and social development of producer countries. The process of growing coffee plants is based on a constant and balanced supply of nutrients to each part of the tree. A poor distribution of nutrients may cause different diseases, such as chlorosis and deformation of leaves, among others, which directly affects the production. Colombia, one of the most important coffee growers in the world, has increased its production in around 10% during last year up to total production, in 2016, estimated in 4.2 millions of bags of processed coffee [9]. However, the negative effects of “el Niño” phenomenon on the production are becoming more evident. In view of this, there exists a need for implementing new strategies and technologies in order to mitigate the effect of long periods of drought by means of a proper control of the water status of the plantations. Furthermore, the measurement of plant hydration is a very important factor which may have implications on fertilization, presence of weeds and the identification of seasons, i.e., when the environmental conditions can produce either excess or defect of water content. This governs the dynamics of flowering and fruit growth, as well as the presence of plagues and diseases [10]. Irrigation control is pivotal as it can be used to increase planting density, to increase crop yield, and to affect fertilization management [11,12,13,14].

Traditionally, the establishment of the amount of water needed to irrigate a crop has been solved by using climatic mean values (potential evapotranspiration or recently, crop evapotranspiration), and by monitoring either the soil water content or plant water status. The first method does not consider inter-annual variations, commonly found in semi-arid regions like the Mediterranean. In the second one, the size and development of the plant root system constitutes a limitation in the calculation of the irrigation requirement of the plant due to the spatial variations of the soil water availability. Therefore, the direct monitor of the plant water status is the only way to accurately adjust the water dose required by the crop. Methods to obtain plant water status are mainly based on the measurement of water potential or relative water content [15,16,17]. Water potential (Ψ) describes the energy status of water in plants, it is expressed as potential energy per unit volume and its units are those of pressure, MPa or bars. The most widely used method to measure Ψ is the so called Scholander pressure-chamber technique. On the other hand, the relative water content (RWC) is the amount of water per unit weight of water at full hydration. The calculation of RWC is based on the following ratio: (fresh weight − dry weight)/(saturated weight − dry weight) [18]. These methods are considered destructive techniques precluding repetitive measurements in a given tissue and, therefore, they are not suitable for studying dynamic water changes within the same plant tissue or organ. For this reason, and during the past decades, there has been a challenge to find non-destructive or non-invasive techniques [19,20,21,22,23,24].

Resonant ultrasonic spectroscopy (RUS) [25] is a well know technique to obtain the elastic constants of solid materials from the analysis of the resonant frequencies of different modes of vibration of samples having a well defined geometry and free boundary conditions [26,27,28]. In a similar way, and for the case of plates, air-coupled ultrasound has been used to excite and sense thickness resonances with a similar purpose: to obtain elastic constants [29,30,31,32,33,34]. In this sense, this technique can be considered as non-contact RUS (NC-RUS), though there are significant differences with conventional RUS (e.g., no free boundary conditions are considered in this case). NC-RUS has also been applied to excite and sense thickness resonances in plant leaves and to determine some of their properties (thickness, density, elastic modulus, mechanical damping) [35]. Moreover, it has been demonstrated that there is a close relation between the parameters extracted from the ultrasonic resonance of the leaves and their relative water content and water potential. In particular, as leaves become dehydrated the variation in the frequency of the first thickness resonance ($f_{res}$) with the relative water content follows a decreasing sigmoid whose point of inflection is located at the turgor loss point [36,37,38,39]. More recently NC-RUS has also been proven as a technique for the dynamic determination of leaf water status [40]. So far, we have applied the NC-RUS technique to more than 50 plant species, where the only requirements is that the leaf surface must be larger than the acoustic beam width and that leaf surface must be relatively flat over the section defined by the acoustic beam width.

The purpose of this paper is to review the main requirements of a NC-RUS sensing system to measure thickness resonances in plant leaves, to propose two different transducer/sensor solutions for two particular cases: *Vitis vinifera* and *Coffea arabica* leaves and to test the possibilities of the proposed solution to determine leaf parameters, RWC and Ψ both in lab and field conditions. Moreover, this same solution can be used for other species whose leaves present thickness resonances in these frequency bands

## 2. Description of a NC-RUS System for Plant Leaves and Main Design Parameters and Specifications

**Figure 1:**
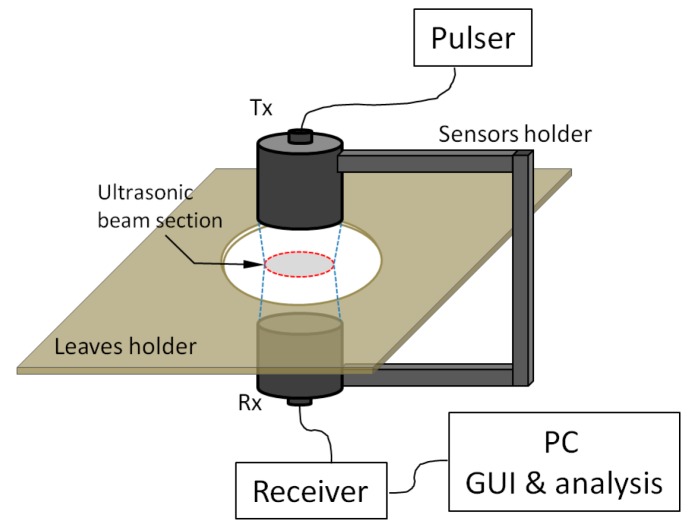
Schematic representation of the NC-RUS system.

Figure 1 shows a schematic representation of the main elements of a NC-RUS system to measure thickness resonances in plant leaves. These elements can be grouped in four categories (sensors, electronics, software and structural elements):

1. Sensors. A couple of wideband and high sensitivity air-coupled ultrasonic transducers (transmitter: Tx and receiver: Rx).
2. Electronics. A pulser/receiver to excite Tx and to filter, amplify and digitize the electrical signal in Rx. If an analog pulser/receiver is used, then a digital oscilloscope or a similar device is required to digitize the received signal.
3. PC and software. Including: (i) software to control the electronics and display the results, includes a graphical user interface (GUI) and (ii) the software to solve the inverse problem and extract leaf parameters from the measured resonance.
4. Structural elements. Including: (i) a system to hold sensors in the right position, (ii) a sample holder that allows the right location of the leaf in-between the ultrasonic sensors and (iii) any system to isolate the measurements from the influence of environmental conditions.

The main design parameters of an NC-RUS system to measure thickness resonances in plant leaves, the elements affected and the specifications to be met are summarized in Table 1.

### 2.1. Size of the Measurement Area and Geometry of the Ultrasonic Field (Beam)

**Figure 2:**
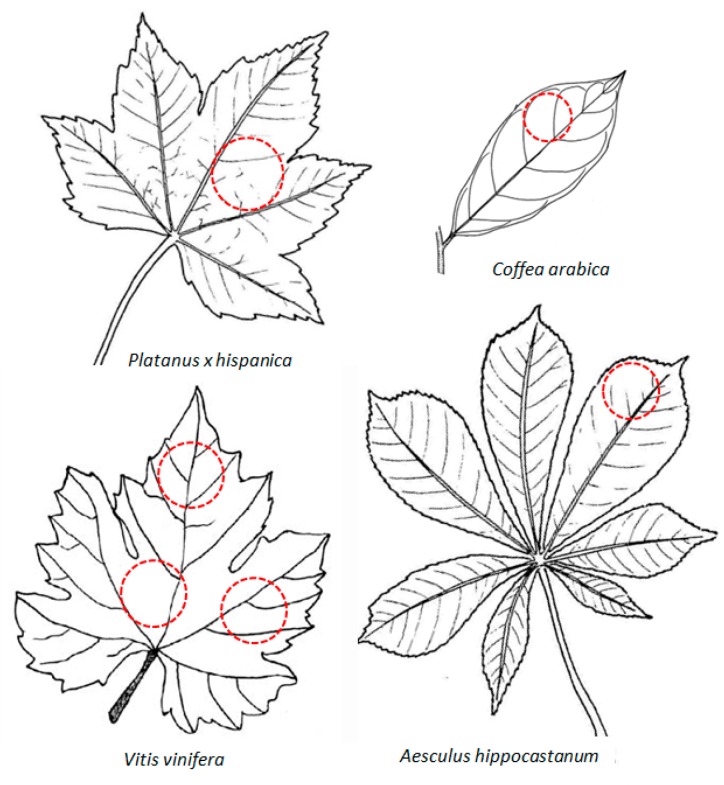
Examples of some leaves and size of the ultrasonic beam and location of the measurements.

Size of the leaf area where measurements are performed coincides with the ultrasonic beam section (see Figure 1) and the size of the beam section is slightly smaller than the size of the transducers aperture (depending on the transducer-leaf distance). As the beam section must be completely included within the leaf, this imposes an upper limit for transducers size. In addition, as obtained leaf properties are averaged values over the measurement area, it is then convenient to take the largest section possible while avoiding any major inhomogeneity like large veins or largely curved parts. As an example, Figure 2 shows acceptable beam size and location point of the measurement area for a few examples.

It must be also be considered that the analysis of the spectra of the thickness resonances is performed assuming plane wave and normal incidence. Therefore, to achieve a wavefront of the incident acoustic beam on the leaf surface as plane as possible, the transducer surface must present a piston like vibration.

### 2.2. Centre Frequency and Frequency Bandwidth

The main requirement for frequency band of the NC-RUS system for the study of the leaves of a given species is that this band must include the whole spectra of the first thickness resonance for all leaves of this species. In general, the leaf resonance spectra (magnitude and phase) are well defined by taking a frequency band or window defined by: Magnitude spectrum peak value—6 dB. In addition, as the value of $f_{res}$ not only varies from leaf to leaf, but is also variable for a given leaf (depends on the degree of development, the water content, etc.), then, the frequency band of the NC-RUS system must be large enough to include all these variations.

**Figure 3:**
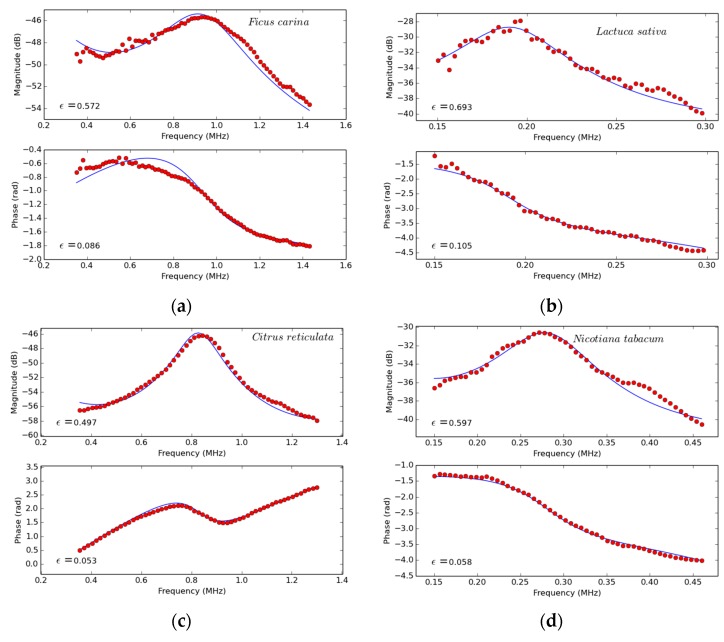

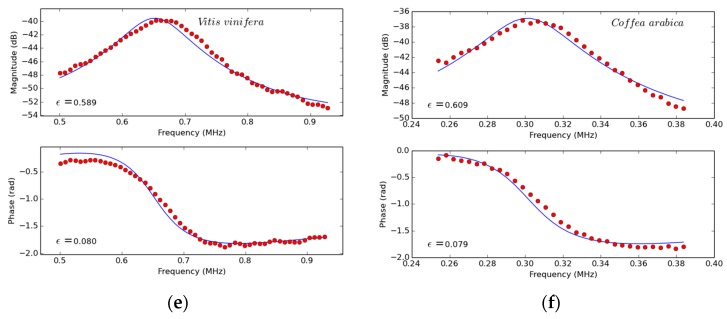
Spectra (magnitude and phase) of the first thickness resonance of plant leaves of different species. Dots: measured values. Solid line: theoretically calculated values assuming a homogeneous layer and using the layer parameters obtained from the solution of the inverse problem. ε is the root mean square deviation of the calculated spectra respect to the measured ones. (**a**) *Ficus carina*; (**b**) *Lactuca*; (**c**) *Citrus reticulata*; (**d**) *Nicotiana tabacum*; (**e**) *Vitis vinifera*; (**f**) *Coffea arabica*.

With the purpose of illustrating the typical requirements, Figure 3 presents some spectra of the first thickness resonance of some leaves of different species that are rather representative of the different situations found. Measurements and theoretical calculations are obtained following the procedure explained in [33,34,35,36,37,38,39]. $f_{res}$ is normally located within the frequency range 0.1–1.0 MHz, where the lower values normally correspond to soft leaves of herbaceous species like *Arabidopsis thaliana* or *Lactuca sativa*. The 6dB relative bandwidth of the resonances observed in Figure 3 is about 70% for the cases where the resonance peak is strongly attenuated (like in *Ficus carina* and *Nicotiana tabacum*), and between 25% and 35% for those cases where the resonance peak is less attenuated (*Coffea arabica*, *Vitis vinifera* and *Citrus reticulata*).

**Figure 4:**
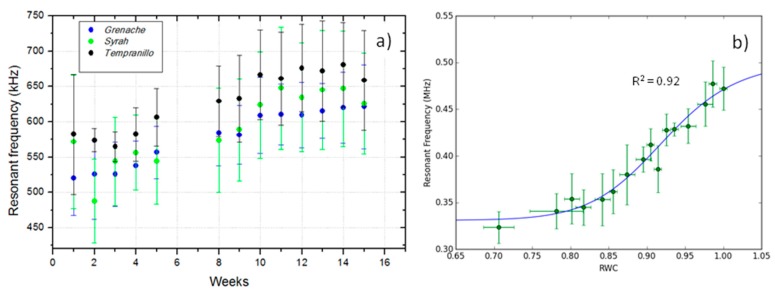
(**a**): Variation in $f_{res}$ for three different *Vitis vinifera* cultivars during the period 1 July–9 October. Week 0 corresponds to the first week of July; (**b**): Variation in $f_{res}$ of *Viburnum tinus* leaves with RWC and fitting of the logistic function into the experimental data (R^2^ of the fitting is on the figure).

As an example of the typical variability of $f_{res}$ from leaf to leaf (with all leaves under similar conditions) measurements in 30 leaves of *Viburnum tinus* and *Arabidopsis thaliana* were performed. The obtained relative variation in $f_{res}$ was 6% (490 ± 30 kHz) and 8% (157 ± 13 kHz), respectively. This range of variation can be considered representative of the behaviour of most of the species. To illustrate the magnitude of the variation in $f_{res}$ with the degree of leaf development, leaves of three different *Vitis vinifera* cultivars planted in 10l pots at CSIC-Madrid were measured in the period May–September. Results are shown in Figure 4a. This variation is due to the change in both the leaves thickness and in the cell wall elastic modulus. Finally, as an example of how RWC affects $f_{res}$, Figure 4b shows some result obtained for *Viburnum tinus* leaves: when RCW decreases from 1.0 to 0.7, relative variation in $f_{res}$ is 33%. Similar results were found for *Coffea arabica* (relative variation in $f_{res}$ of 26%) and *Vitis vinifera* (relative variation in $f_{res}$ of 20%). All these variations must be taken into account in the design of the transducers for NC-RUS for a given species.

### 2.3. Dynamic Range and SNR

Though transmission loss at resonance (see Figure 3) is typically between 35 and 45 dB, smaller figures are obtained for herbaceous leaves (between 25 and 32 dB). In most cases, the spectrum of the first thickness resonance is well defined by taking a frequency band around the thickness resonance given by 6 dB loss respect to the peak value. This means that the minimum value of the modulus of the transmission coefficient to be measured is >−60 dB.

### 2.4. Separation between the Sensors and the Leaf

Separation between transducers and leaf (∆L) must be large enough so that the through transmitted signal does not overlap with the reverberations in the transducer/leaf air-cavity. Therefore the time for the ultrasonic signal to cross twice the distance between transducer and the leaf (∆t) must be larger than the duration of the through transmitted pulse (δt). δt depends on the centre frequency and bandwidth of both the transducers and the leaf thickness resonance. Typically, δt < 40 μs, then: ∆L > 14 mm. On the other hand, separation between transducers and leaf must be kept as short as possible to minimize the attenuation in the air, and any possible interference in the air path. To minimize the size of the beam section on the leaf surface, the leaf can be located at the natural focal length of the transducers which is located at *a^2^/λ*, where *a* is the radius of the transducer aperture and λ is the wavelength of the radiated beam.

### 2.5. Time of Measurement

The time to take one measurement must be small enough to allow for fast and in situ measurements. Given that the separation between Tx and Rx is normally smaller than 60 mm, the time to take one measurements is smaller than 180 μs. If several signals are to be acquired to take an average and improve SNR, then this time will be increased. Time to take this averaged measurement will then be mainly determined by the pulse repetition frequency (PRF) of the pulser/receiver and the number of samples to average. PRF values between 100 and 1000 Hz and averaging between 10 and 100 samples are normally used, this implies that the elapsed time will be between 1 and 0.1 s, respectively. However, the most time consuming stage will be the processing of the signal and the solution of the inverse problem to extract leaf parameters. Time of execution of the inverse problem code can be reduced by reducing the length of the digitized resonance spectra. For lengths below 100 points and inverse solution codes written using, relatively low speed, interpreted languages (like Matlab or Python) it is possible to obtain execution times below 10 s, which is quite acceptable for this application.

### 2.6. Portability and Robustness

Portability requirements for lab measurements are reduced; however, this is not the case for field applications. In these cases, the PC must be a laptop or a tablet, the electronics must be powered by batteries and the sensors must be embedded on a portable holder. The most demanding robustness requirements also correspond to field measurements as the influence of the environmental factors on the measurement must be reduced. In particular, an easy way to locate the leaf between transducers and some protection against possible strong winds must be provided. In addition, resistance of sensors to air moisture and temperature must be also considered.

## 3. Proposed NC-RUS System for Plant Leaves: General Solution

### 3.1. Sensors

The active element is always a 1–3 connectivity piezoelectric composite disk made of piezoceramic pillars embedded in an epoxy matrix, instead of bulk piezoceramics commonly used in other applications. This selection is determined with the purpose of reducing the presence of radial or lateral vibration modes in the piezoelectric component of the sensors so that active area can be reduced while keeping a piston like vibration. In addition, 1–3 connectivity piezocomposites present some additional benefits as larger bandwidths and lower acoustic impedance values compared with bulk ceramics can be achieved, which permits to produce air-coupled transducers with larger sensitivities and bandwidths.

**Figure 5:**
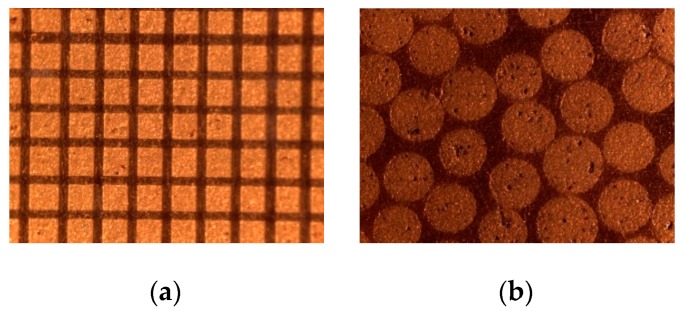
Microscopic image of the piezocomposite surface. (**a**): Dice and fill composite with 130 μm square ceramic pillars, 48 μm pitch and 70% ceramic volume fraction; (**b**): Random ceramic fibers composite with 250 μm fiber diameter and 65% ceramic volume fraction.

There are two main commercially available solutions (see Figure 5): (i) Dice and fill composites and (ii) composites made of ceramic fibers (either random or regular distribution) embedded in a polymeric matrix. In this case, the second solution was used, with PZT5A 250 μm diameter fibers randomly distributed in an epoxy resin matrix. Ceramic volume fraction is 65%.

Impedance matching to the air follows the basic criteria and materials proposed in references [41,42] where the acoustic impedance of the outer matching layer is always about 0.04 MRayl. A backing block is added to improve the transducers frequency band. The transducer housing is made of anodized aluminum to provide EM shield as well as resistance to environmental moisture.

**Figure 6:**
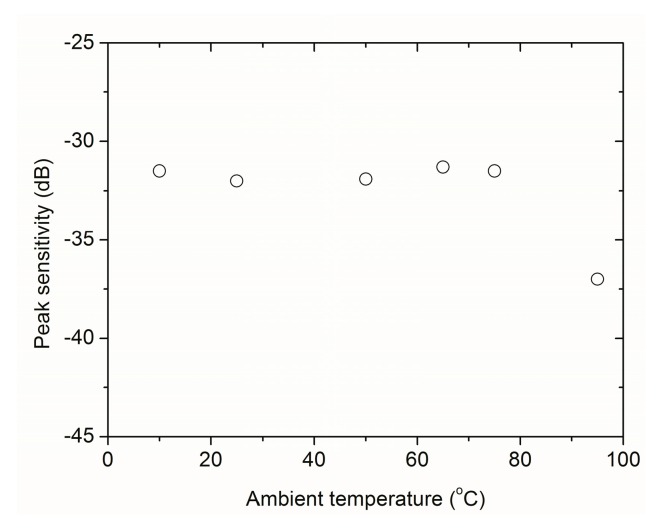
Variation in the peak sensitivity of a pair of air-coupled transducers (1 MHz) with the ambient temperature.

The robustness of this design has also been tested. The major potential effect of environmental moisture is on the radiating surface that is made of a porous layer. Three solutions have been successfully implemented: (i) use of hidrophopic porous materials (ii) use of closed cell porous materials (iii) protecting the surface with an impervious very thin layer. In this later case, either parylene coatings or spin coated PMMA have been used when pore size is smaller than 0.1 μm. Figure 6, shows the variation in the peak sensitivity with the temperature of a pair of 1 MHz air-coupled transducers fabricated with this technology, response is rather flat up to 80 °C.

### 3.2. Mechanical Holder for Sensors and Leaf

**Figure 7:**
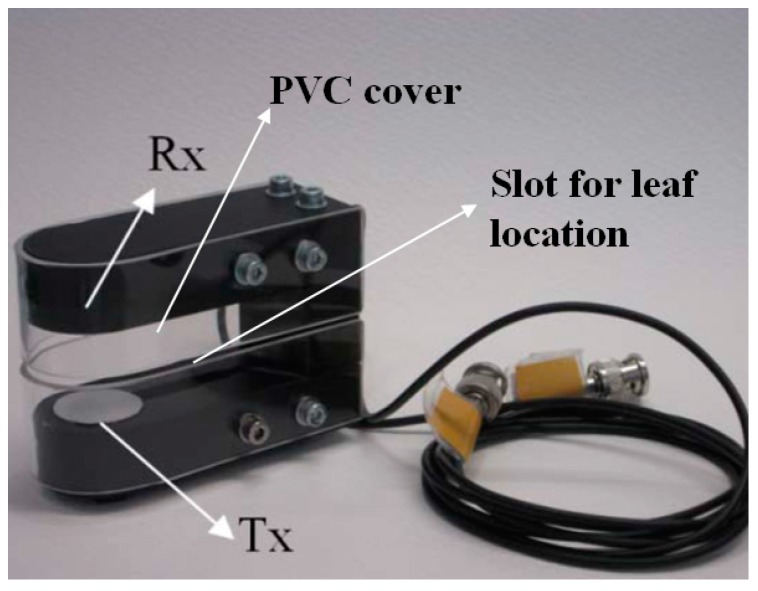
Picture of the mechanical holder for sensors and leaf.

An U-shaped holder for the Tx and the Rx is proposed (Figure 7). This system permits to locate the sensors in the right position and at the right distance, while permits to move the whole system without altering the relative position of the sensors. In addition a PVC cover provides protection Against environmental factors (water and wind) as well as a slot for the right location of the leaf for the measurements.

### 3.3. Electronics

Frequency bandwidth of both pulser and receiver stages must cover the whole frequency range for the applications considered. Pulse amplitude and gain in reception must be large enough to make possible to measure the transmitted signal through the leaves with a good SNR figure. Typical figures for this application are: (i) electrical pulse excitation amplitude: 200–400 V; (ii) shape of the excitation electrical pulse: either spike or semicycle of square wave; (iii) gain at reception between 30 and 40 dB. These values are quite normal for most commercial ultrasonic equipments, in particular, we have successfully used the following pulser/receivers: Olympus 5077 and 5058, DASEL Ultrascope and Lecoeur electronics USB-key. The later one only for leaves with $f_{res}$ larger than 0.5 MHz because of limitations in the pulser/receiver frequency band.

### 3.4. Control Software (GUI)

In the case of using analog electronics (Olympus 5077 and 5058), the received signal is digitized by a digital oscilloscope (Tektronix). The process to acquire the signals, store data and perform calculations is controlled by MATLAB. In the case of using digital electronics (DASEL and Lecoeur), the control of the pulser/receiver and the display of the results is performed by a GUI designed in Labview.

### 3.5. Data Processing

The extraction of leaf parameters from the measured resonance spectra is performed by solving the inverse problem as explained in references [38,43]. The code is written in Python 2.7 and is available through reference [44]. This code is called either by MATLAB or Labview GUI to perform the fitting after the measurements are taken. The correct fitting of the theoretical curve into the experimental data (some examples appear in Figure 3) is used, firstly, to validate the measurements and, secondly, to extract leaf parameters: thickness, density, ultrasound velocity and ultrasound attenuation coefficient.

### 3.6. System Integration

**Figure 8:**
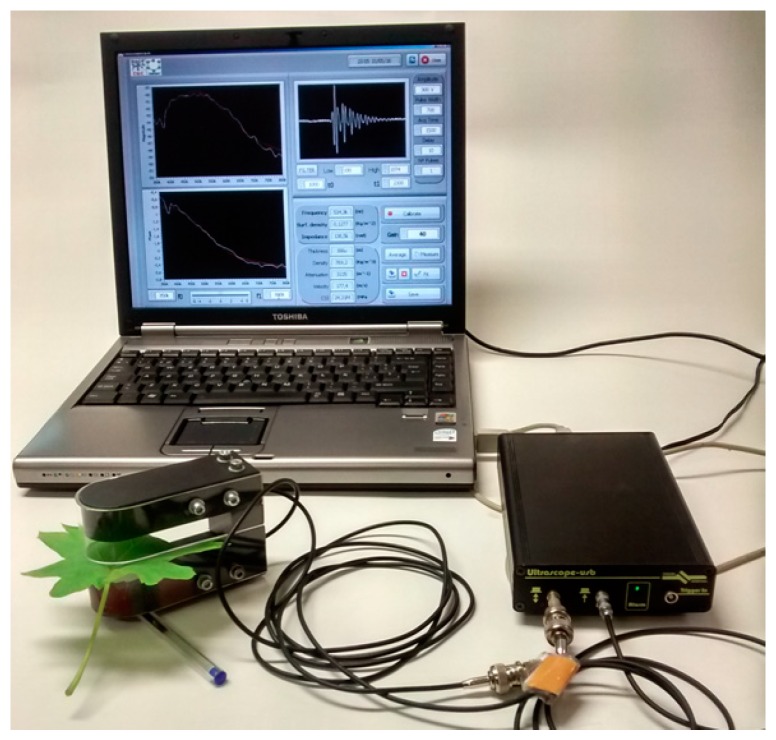
Example of NC-RUS system realization for *Vitis vinifera* leaves.

Figure 8 shows a realization of a portable equipment including a laptop with a Labview GUI, a Dasel Ultrasocope pulser/receiver and the U-shaped transducer holder with a *Vitis vinifera* leaf inserted in the leaf-slot.

## 4. Specifications of the NC-RUS System for *Coffea Arabica* and *Vitis Vinifera* Leaves: Sensors and Structural Elements

Table 2 summarized the main design criteria for the sensors and electronic components of the NC-RUS system for *Vitis vinifera* and *Coffea arabica*.

### 4.1. Specific Design of Sensors for Vitis Vinifera Leaves

**Figure 9:**
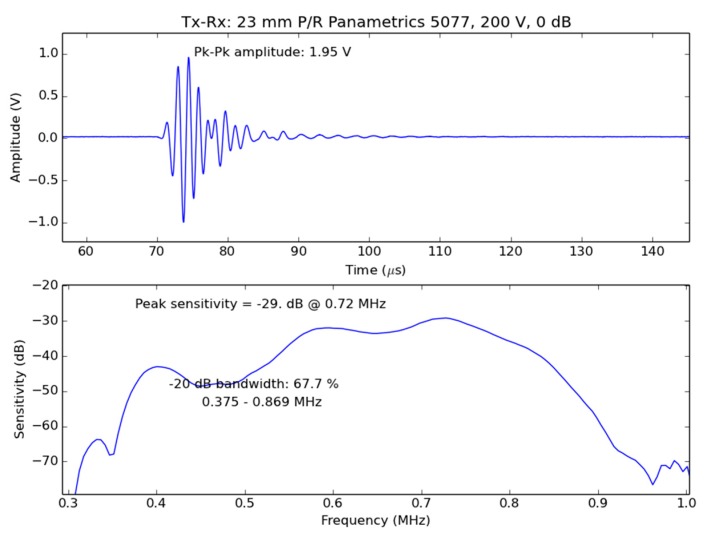
Response of the Tx-Rx pair for *Vitis vinifera* leaves in through transmission. Upper figure: the time domain response. Lower figure: sensitivity band.

To meet the specifications in Table 2, 15 mm diameter piezocomposite disks with resonant frequency at 650 kHz , were used to make the transducers. Matching to the air is achieved by following the method of [41,42]. Transmitter transducer was driven by a Olympus 5077 pulser (amplitude 400 V) and the receiver transducer was connected to the receiver stage of the Olympus 5077 (gain 40 dB). Signal was then transferred to a Tektronix 7054 DPO oscilloscope to digitize the signal, extract FFT and display the results. Response in the time domain and the sensitivity frequency band are shown in Figure 9. Sensitivity is calculated by:

(1) $$SNS\left(dB\right)=20\;log\left(\frac{FFT\left(V_{Rx}\right)}{FFT\left(V_{Tx}\right)}\right)$$

where V_Rx_ and V_Tx_ are the voltages measured at the receiver and transmitter transducers terminals, respectively. Peak sensitivity is −29 dB, SNR 70 dB (16 averaging), centre frequency 600 kHz and the −20 dB relative bandwidth is 68%.

In this case, the duration of the pulse is about 20 μs, separation between transducer and leaf must be >14 mm. Figure 3 shows the measured magnitude and phase spectrum of a Vitis vinifera leaf with the transducers developed for this case (Figure 9), in this case, SNR was 46 dB. The usable frequency range in this case is: 350–950 kHz. At these frequencies, total loss can be obtained from the contribution of transducers sensitivity and leaf insertion loss, that is, 97 and 125 dB, respectively.

### 4.2. Specific Design of Sensors for Coffea Arabica Leaves

**Figure 10:**
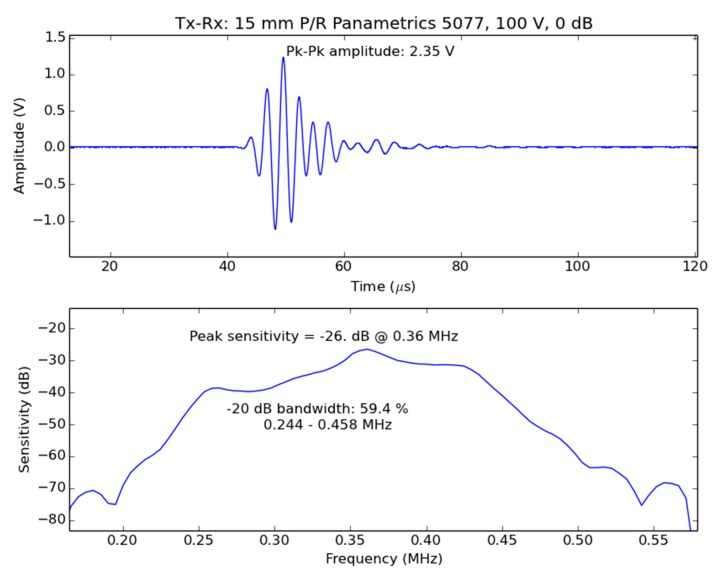
Response of the Tx-Rx pair for *Coffea arabica* leaves in through transmission. Upper figure: time domain response. Lower figure: sensitivity band.

**Figure 11:**
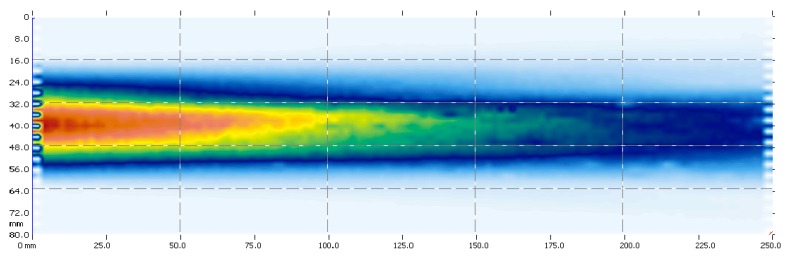
Acoustic field of the transducers for *Coffea arabica*.

The proposed solution to meet specifications in Table 2 is based on a 1–3 connectivity piezocomposite disk with thickness resonant frequency at 350 kHz and diameter 20 mm; matching to the air is performed by following the method proposed in reference [41,42]. Figure 10 shows the measured signal in the receiver transducer when the transmitter transducer is excited with the Olympus 5077 Pulser/receiver (100 V amplitude) and the receiver is connected directly to an oscilloscope (Tektronix 7054 DPO), separation was 13 mm. Peak sensitivity is −26 dB, SNR 63 dB (16 averaging), centre frequency 360 kHz and 59% of −20dB relative bandwidth. As the duration of the pulse is about 35 μs, separation between transducer and leaf must be >24 mm, so separation between transducer must be >48 mm. Figure 11 shows the acoustic field of these transducers. At a distance of 24 mm from the transducer surface (the minimum distance where the leaf can be located for the measurements) the width of the acoustic beam is about 20 mm, which also complies with the imposed requirements.

Figure 3 shows the measured magnitude and phase spectrum of a *Coffea arabica* leaf with the transducers developed for this case (Figure 10 and Figure 11). Transmitter transducer was driven by a Olympus 5077 pulser (amplitude 400 V) and the receiver transducer was connected to the receiver stage of the Olympus 5077 (gain 40 dB). Signal was then transferred to a DPO 7054 Tektronix oscilloscope to digitize the signal, extract FFT and display the results. The measured SNR with the leaf between the transducers was 43 dB and the usable frequency in this case is: 250–540 kHz At these frequencies, total loss can be obtained from the contribution of transducers sensitivity and leaf insertion loss, that is, 83 and 130 dB, respectively.

## 5. Examples of Application

### 5.1. Vitis Vinifera

Three different experiments were performed on *Vitis vinifera* leaves. In the first one, magnitude and phase spectra of the first thickness resonance of a Grenache leaf were used to solve the inverse problem and to extract the leaf parameters. In the other two experiments, we focus only on $f_{res}$ measured directly on the spectrum, as a means to monitor the variation in RWC and Ψ. The Scholander pressure chamber method, which is a reference method in this field, was used to measure Ψ.

#### 5.1.1. Extraction of Leaf Parameters from the Measured Spectra of the First Thickness Resonance

The measured resonance spectra and the theoretically calculated spectra using the extracted leaf parameters obtained by solving the inverse problem is shown in Figure 3. The extracted leaf parameters for the *Vitis vinifera* leaves are shown in Table 3: thickness (*t*), leaf mass per area (LMA), leaf elastic modulus in the thickness direction ($c_{33}$) and attenuation of ultrasonic waves at resonance (α). Thickness and LMA data are comparable with previously published data [45].

#### 5.1.2. Use of NC-RUS to Monitor Drought Stress in *Vitis vinifera*

A well watered five-year-old potted plant of Vitis vinifera cv. Grenache was placed under a transparent greenhouse tunnel of alveolar polycarbonate to avoid re-watering by storms or unwanted rainfall events. Watering was stopped on mid-summer and measurements Ψ and $f_{res}$, using the proposed NC-RUS system, were performed every two or three days with increasing levels of drought stress. Measurements were conducted strictly at predawn (pd) and at 12 h solar time (midday, md). Measurements of $f_{res}$ were performed in the same full developed leaf, while Ψ was obtained from different leaves as this parameter has to be measured with a destructive method (Scholander pressure chamber). Drought stress was imposed during 20 days.

**Figure 12:**
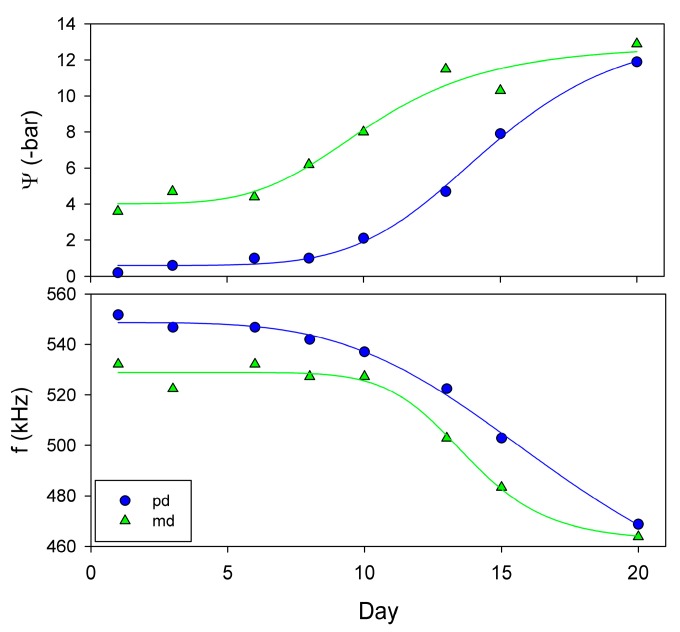
Evolution of Ψ and $f_{res}$ (f) to drought for a single leaf of Vitis vinifera cv. Grenache (circle) measured at predawn (pd, circle) and midday (md, triangle) along the water stress experiment.

The Evolution of Ψ and $f_{res}$ to drought for *V. vinifera* (Figure 12) indicates that when plants became water stressed, Ψ and $f_{res}$ varied simultaneously. The plant started the experiment with predawn values of Ψ and *f* around 0 bar and 550 kHz respectively. Eight days after the last watering, predawn Ψ and $f_{res}$ became slightly lower, reaching values of −1 bar and 542 kHz. From here, these values dropped to c.a. −12 bar and 469 kHz, respectively, at the end of the dry period. Regarding the midday measurements given in Figure 12, their trends are similar to those measured at predawn: a slight decrease of Ψ and $f_{res}$ during the first days of the experiment followed by a drop at the end of the dry period.

#### 5.1.3. Use of NC-RUS to Monitor the Dehydratation Process of *Vitis vinifera* Leaves: Relationship between Resonant Frequency, Water Potential and Relative Water Content

**Figure 13:**
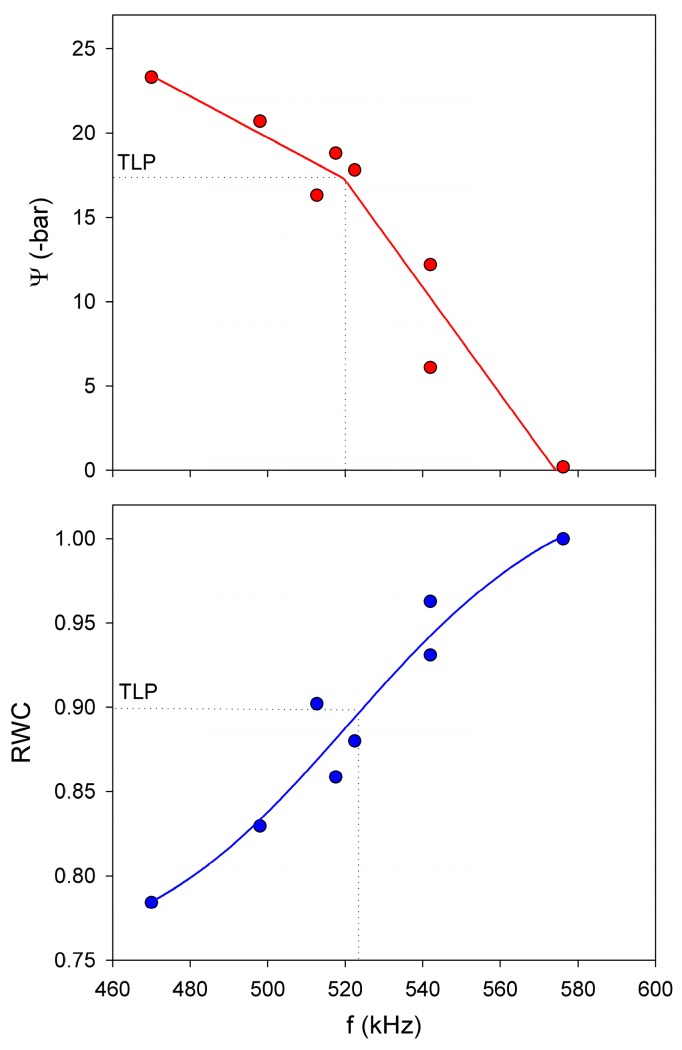
Relationships between $f_{\text{res}}$ (f) with Ψ and RWC for a Vitis vinifera cv. Grenache leaf. Dotted line indicates the turgor loss point (TLP).

Additionally, a third experiment was carried out on a detached single leaf of *V. vinifera* cv. Grenache. This experiment consisted of measuring $f_{res}$, Ψ and leaf weight along a dehydration process following the free transpiration method described in previous studies [46]. Leaf weight was used to calculate the relative water content (RWC) [47]. The values for a single leaf of $f_{res}$ obtained during a dehydration process are represented against different levels of Ψ and RWC in Figure 13. The relationship between Ψ and $f_{res}$ was adjusted to a linear segmented model (R^2^ adj = 0.88), which is characterized by the existence of a join point. In the other hand, the relationship between RWC and $f_{res}$ was adjusted to a four parameter logistic curve (R^2^ adj = 0.88), characterized by the existence of an inflexion point. Both the join point and the inflexion point corresponded statistically with the turgor loss point (TLP), an important physiological moment where the leaf losses its turgor [39,48].

### 5.2. Coffea Arabica

Three different experiments were performed on *Coffea arabica* leaves. In the first one, one spectra of the first thickness resonance of one leaf was used to solve the inverse problem and to extract the leaf parameters. In the other two experiments, the objective was to determine the relationship between the ultrasonic transmission coefficient spectra and RWC and Ψ.

#### 5.2.1. Extraction of Leaf Parameters from the Measured Spectra of the First Thickness Resonance

The measured resonance spectra and the theoretically calculated spectra using the extracted leaf parameters obtained by solving the inverse problem is shown in Figure 3. The extracted leaf parameters for the *Coffea arabica* leaves are shown in Table 3. Thickness and LMA results are comparable with previously published data [49].

#### 5.2.2. Extraction of Leaf Parameters from the Measured Spectra of the First Thickness Resonance

The starting point was fully hydrated *Coffea arabica* leaves. The plant leaf samples were cut by its petiole and kept, in a refrigerator, inside plastic bags filled with water during 12 h. Then, using the proposed NC-RUS system, the insertion loss coefficient is computed at different dehydration levels, which are obtained by consecutively placing the sample inside the chamber of a precision moisture balance (MA60.3Y, Radwag, Poland) at 30 °C during 10 min per dehydration interval. Each time the sample is taken out from the dehydration chamber, NC-RUS measurements are carried out at four different zones of the leaf, aiming to get information about the global water content of the sample. In addition, at each step, the corresponding weight is recorded. Using the dry weight of the sample, the RWC is calculated and the relationship with NC-RUS spectra is then obtained.

**Figure 14:**
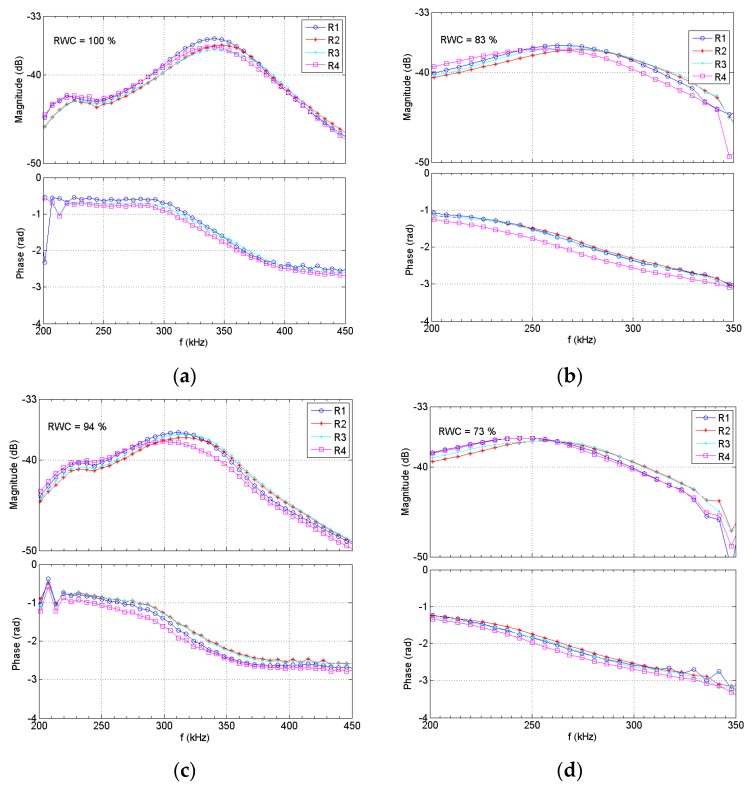
Measured spectra in a *Coffea arabica* leaf at four different positions (R1, R2, R3 and R4) and at four different RWC values (100%, 94%, 83% and 73%). (**a**) RWC = 100%; (**b**) RWC = 83%; (**c**) RWC = 94%; (**d**) RWC = 73%.

**Figure 15:**
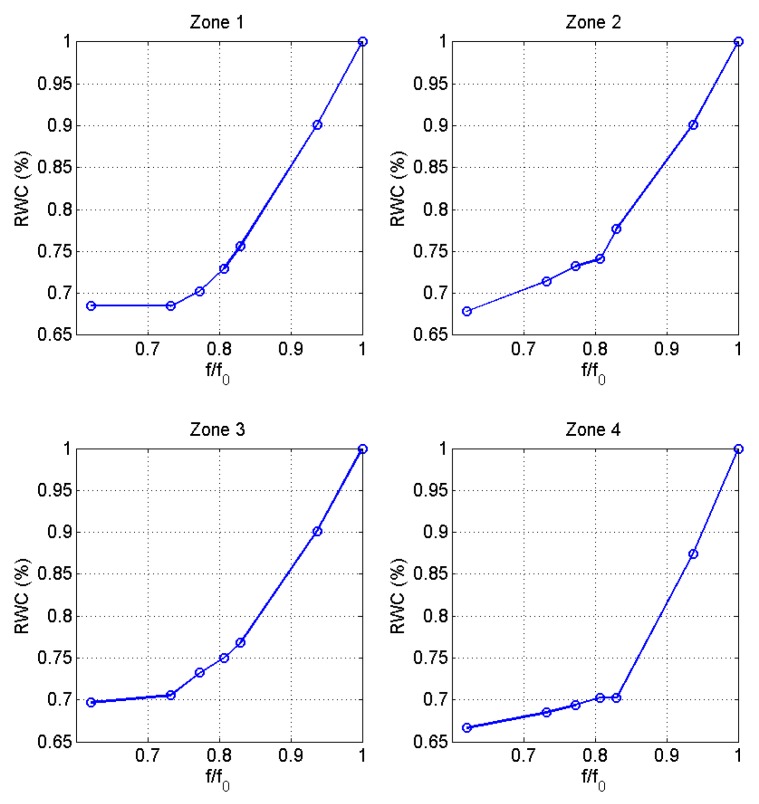
Variation in RWC as a function of the normalized resonance frequency in four different zones (R1, R2, R3 and R4, respectively) of the leaf.

Figure 14 shows the magnitude and phase spectra obtained by the proposed NC-RUS system for 4 hydration levels (RWC = 100%, 94%, 83% and 73%), of a *Coffea arabica* leaf, measured in four different zones of the leaf (R1, R2, R3 and R4). It is appreciated, that $f_{res}$ and phase at resonance decrease as RWC diminishes. Figure 15 shows RWC as a function of the normalized $f_{res}$ of the leaves progressively dehydrated. Every point in the plots stands for the RWC on the respect measured region of the sample. *f_0_* is the resonance frequency at full hydration (saturation state), and f is the respective $f_{res}$ at every hydration level.

#### 5.2.3. Relationship between Ultrasonic Transmission Spectra and the Water Potential

The experimental relationship between the NC-RUS spectra and water potential is obtained by modifying the whole water status of a *Coffea arabica* plant in the following way: First, the plant under test is left without irrigation during two days. To begin, early in the morning, the water potential is measured from different leaves of the plant under test using a Scholander Pressure bomb (Model 1505D, PMS Instrument Company, Albany, OR, USA). Almost simultaneously, NC-RUS spectra in four different zones of the leaves removed from the plant are captured. In addition, the weight of each leaf sample is recorded. Then, the plant is irrigated with 2.5 L of water at ambient temperature at intervals of 2 h. Before each irrigation step, we obtained the NC-RUS spectra, water potential and weight of a new set of 3 new leaves. Two irrigation steps were carried out.

**Figure 16:**
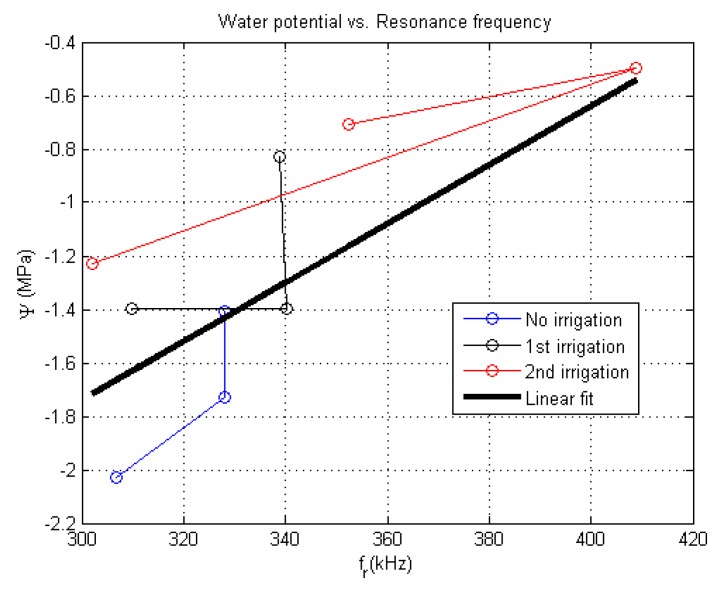
Water potential as function of ultrasonic resonance frequency obtained for 9 different leaves of *Coffea arabica* under 3 different states of hydration. A linear fit is also included (R = 0.745).

Figure 16 shows the averaged resultant correlation between measured water potential and ultrasonic resonance frequency obtained for different leaves of *Coffea arabica* under different states of hydration. Apart from the point most to the right (fr > 400 kHz), the resonant values fell within the frequency range shown in Figure 14, i.e., water potentials below −0.8 MPa correspond with resonant frequencies between 300 and 340 kHz. The data points obtained show an uphill trend as the irrigation is increased. The straight lines connecting the point were included to account for the three different irrigation states of the plant under test. It is observed that the point most to the left, at the second irrigation step, seems no to follow the trend. By removing this point, the correlation coefficient R is 0.811 for a linear fitting. With no points removed, R is 0.745. Regarding the observed resonance frequency at 408 kHz (the point most to the right), this could be attributable to the fact that we were measuring on a particular leave with either increased mechanical properties or a thinner thickness.

## 6. Conclusions

Main specifications for an NC-RUS system, including sensors, electronics, software and structural elements, to measure plant leaves have been reviewed. In particular, requirements for air-coupled ultrasonic transducers for NC-RUS measurements in *Coffea arabica* and *Vitis vinifera* leaves have been determined. Following these design criteria, transducers were produced using 1–3 connectivity piezocomposites and matching to the air as described in [41,42]. For *Coffea arabica* leaves, centre frequency of the transducers is located at 350 kHz with a peak sensitivity of −26 dB and the operation bandwidth covers the frequency range 200–450 kHz, which corresponds to the frequency band 20 dB below the main peak. For *Vitis vinifera* leaves, the centre frequency is located at 650 kHz with a peak sensitivity value of −29 dB. The useful bandwidth covers the frequency range 350–900 kHz. Using these transducers and commercially available and general purpose electronic equipment to drive the transmitter transducer (400 V amplitude semicycle of square wave) and to amplify the received signal (+40 dB) it has been possible to measure *Coffea arabica* and *Vitis vinifera* leaves under different conditions and to establish a relationship between $f_{res}$, RWC and Ψ which confirm the possibility to use this technique to obtain accurate information of the crop irrigation needs. For *Vitis vinifera* detached leaves $f_{res}$ decreases from 580 kHz to 460 kHz when Ψ varies from 0 bar (RWC = 1) to −25 bar (RWC = 0.78). In addition, in vivo measurements on trees subjected to water stress (20 days drought) revealed that variations in the predawn and midday $f_{res}$ values were consistent with the variations observed in Ψ. Predawn $f_{res}$ varies from 550 kHz (day 0) to 470 kHz (day 20), while the predawn Ψ varies from 0 bar (day 0) to −12 bar (day 20). In detached *Coffea arabica* leaves, $f_{res}$ is about 350 kHz (RWC = 1) and decreases to 225 kHz at RWC = 0.7. The unique ability of the proposed NC-RUS system to register changes of the plant water status under conditions of free leaf transpiration constitutes a tool of paramount importance in order to maximize water use efficiency in crop plants. Applying this ultrasonic system in agriculture, water consumption could decrease by adjusting the irrigation doses to the plant water necessity. The adjustment of irrigation doses by NC-RUS on *V. vinifera* and on *C. arabica* could avoid both scarce watering, that could decrease production, and generous watering, that could reduce quality.

## Figures and Tables

**Table 1:** Summary of the main design criteria and elements of the NC-RUS system affected.

Design Parameter	Elements of the NC-RUS System Affected	Goal
1. Size of the measurement area	Sensors	Smaller than leaf half width
2. Geometry of the ultrasonic field (beam)	Sensors	Plane wave incident on the leaf surface
3. Centre frequency	Sensors and electronics	6 dB band of the thickness resonance band of the leaves to be studied (and expected range of variation) must be included within the frequency band of the NC-RUS system
4. Frequency band	Sensors and electronics	
5. Dynamic range and SNR	Sensors and electronics	Large enough to cope with expected losses for the leaves of the species of interest.
6. Separation between sensors and leaf	Sensors holder	To avoid any overlap of the through transmitted signal with reverberations in the air cavities between transducers and samples.
7. Time of measurement.	PC and software analysis	Fast enough to permit processing measurements right after the acquisition.
8. Portability and robustness	All	To allow for field measurements. Resistance against environmental conditions (wind, moisture, heat, etc.)

**Table 2:** Design parameters for NC-RUS sensors for *Vitis vinifera* and *Coffea arabica*.

Species	Diameter of the Beam	Centre Frequency (kHz)	Frequency Band (kHz)	SNR * (dB)
*Vitis vinifera*	<25 mm	650	400–900	>65
*Coffea arabica*	<20 mm	300	200–400	>60

*: No leaf between transmitter and receiver.

**Table 3:** Extracted leaf parameters from thickness resonance spectra in Figure 3 for *Vitis vinifera* and *Coffea arabica* leaves.

Species	*T* (μm)	LMA (g/m^2^)	$c_{33}$ (MPa)	*α* (Np/m)
*Vitis vinifera*	174	220	68	1625
*Coffea arabica*	210	153	12	1070
